# The root signals in rhizospheric inter-organismal communications

**DOI:** 10.3389/fpls.2022.1064058

**Published:** 2022-12-21

**Authors:** Dongmei Lyu, Donald L. Smith

**Affiliations:** Department of Plant Science, McGill University, Sainte Anne de Bellevue, QC, Canada

**Keywords:** root exudate, signaling, rhizomicrobiome, holobiont, plant-microbes interaction, C-fixation

## Abstract

Root exudates play a key role in mediating plant–plant and plant–rhizomicrobiome interactions, including regulating biochemical/physiological aspects of plant-associated microorganisms, to enhance host plant growth and resilience. Root exudates can act as signals to reduce the competition from neighboring plants and recruiting/choreographing a wide range of diverse rhizomicrobiome members to make the host plant a good fit with its immediate environment. Root exudate production is a dynamic and key process, but there is a limited understanding of the metabolites or metabolic pathways involved in the inter-organismal communications facilitated by them. Given the well-known symbiotic relationships between plants and associated rhizomicrobiome members, adding root exudates to microbial isolation media may allow some of the large segments of rhizomicrobiome members that are not currently culturable to be grown *in vitro*. This will provide new insights into how root signals orchestrate associated microbes, will benefit agricultural production in the face of challenges posed by climate change, and will help to sustainably provide food for a growing global human population.

## Highlights

- Root exudate is part of the plant holobiont; its production varies with the surrounding circumstances.
- As a key aspect of root exudates, signal exchange is responsible for inter-organismal interactions such as plant–microbe, plant–kin, and neighbors in the rhizosphere and within the ecosystem.
- The combination of the advanced omics techniques is a promising approach to deeply explore and exploit the dynamics and abundance of root exudates.
- Plants recruit beneficial microbes through exudates to facilitate positive relationships and enhance plant development and resilience; specific elements of root exudates may also be required to allow growth of many phytomicrobiome members.

## 1 Root exudates

Root exudates contain a wide range of organic compounds including organic acids, sugars, and many other metabolites, which are secreted by plants into the rhizosphere (Baetz and Martinoia, 2014; Preece and Peñuelas, 2020; Vives-Peris et al., 2020) *via* diffusion, ion channel pumping, and vesicle transport (Vickers, 2017; Demidchik, 2018; He et al., 2021). Its production is generally considered to result in one of the most complex ecosystems, which is affected by both aboveground processes and conditions, and the belowground surrounding environment (Korenblum et al., 2020). Light (intensity, wavelength, and photoperiod) is among the most important aboveground factors affecting root exudation, as it directly affects CO_2_ fixation through photosynthesis, which determines the amount of material available for root secretion into the rhizosphere (Vives-Peris et al., 2020). Ten percent to 50% of photosynthetically fixed carbon is translocated into the root and released into the spaces between cells and the root-associated soil (Korenblum et al., 2020). Root exudation patterns are also correlated with functional traits of specific plants (Herz et al., 2018). More leaf area or taller canopies are closely linked to plant relative growth rate including the amount of root produced, which thus could contribute to the secretion of larger amounts of root exudates (Herz et al., 2018).

The belowground response to the root exudate composition mainly depends on soil conditions including nutrient availability and the rhizomicrobiome in rhizosphere (Korenblum et al., 2020; Gao et al., 2021; Jiang et al., 2022; Wen et al., 2022). Plants can shift exudation levels of specific metabolites to address phosphate deficiency stress for both domesticated maize (*Zea mays* subsp. *mays*) and wild species (*Z. mays* subsp. *parviglumis*) (Brisson et al., 2022). A similar response also happened for other plant species, such as maize (Zhengdan 958) under the nitrogen supply (Hao et al., 2022), pearl millet genotypes (sensitive (843-22B) under drought stress (Ghatak et al., 2022), and species from *Curcurbitaceae* family under salinity (Li et al., 2021). In one important aspect, all the above studies also noticed the alteration of rhizosphere microbial communities. As previously reported, maize cultivar *Zman2* released less dolabralexins, which led to more diverse root microbiome, compared with its wild-type (WT) sibling, a cultivar with highly abundant dolabralexins in roots (Murphy et al., 2021). The number of bacterial genera significantly correlated with the level of the root-released organic carbon released by wheat (*Triticum aestivum L.*) (Chen et al., 2019). A subset of bacterial and fungal taxa were enriched under low phosphate, while another set of taxa were abundant under high-level phosphate conditions (Brisson et al., 2022). The quantity and quality of plant root exudates produced are also a function of plant genetics, which have profound effects on modulation of the rhizomicrobiome (Vives-Peris et al., 2020). Wippel et al. (2021) found that host preference in commensal bacteria from different taxa was related to their invasion of standing root-associated communities after comparing the root microbiota under different growth patterns among *Lotus* and *Arabidopsis*.

Plant roots coordinate the aboveground (leaf and shoot) and belowground (microbiota in the rhizosphere) elements of the holobiont, thus regulating and balancing the critical physiological functions in the microbiota–root–shoot environment (Hou et al., 2021). Organic compounds in root exudates can enhance nutrient supply to the soil/root microbiota (Sasse et al., 2018), which contributes to plant growth and soil health. In addition to being a carbon source, root exudates can also mediate biological activity such as nutrient cycling and the dynamics of soil-borne pathogen development (Coskun et al., 2017). Pathogenic microbes in soil can trigger plant immunity by modulating the root metabolism (resulting in specific exudate profiles) to recruit the root microbiota most effective in promoting plant defense and resistance to pest organisms (Berendsen et al., 2018; Yuan et al., 2018; Hou et al., 2020). Root exudates also conduct elements of communication between plants and associated microorganisms, and with neighboring plants (Preece and Peñuelas, 2020), including shaping the composition of the rhizomicrobiome and reducing inter-plant competition *via* allelopathy exudates (Calvo et al., 2017; Vives-Peris et al., 2017; Canarini et al., 2019; Wang et al., 2019; Jacoby et al., 2020; Tian et al., 2020). These communications/interactions among holobiont members are mediated through root exudates, which can act as signal compounds, establishing mutually beneficial relationships and making the host plant well adapted to its immediate environment.

While we know that root exudates have multiple functions, the inaccessibility of the root system has made it difficult to collect and characterize root exudates, resulting in limited understanding of the detailed mechanism(s) involved. In this paper, the role of root exudates in rhizospheric inter-organismal communications is introduced, including collection approaches and analysis techniques applied to root exudates, followed by a potential combination between root exudates and currently unculturable rhizomicrobiome members *in vitro*, finishing with considerations regarding future research required to understand the role of root exudates and their application to enhancement of agricultural productivity.

## 2 Signal exchange

The rhizosphere is a hot spot of information transfer in microbe–plant, microbe–microbe, and plant–plant interactions, *via* signals including phytohormones and other secondary metabolites (Wang et al., 2020) from root exudates. Such communications/interactions are always accompanied by the signals released by plants, and in some cases by both plants and microbes. A signal produced by the plant can induce a return signal released by a microbe, or inhibit microbial signal production, so that it can be both positive and negative in nature (Buhian and Bensmihen, 2018; Lyu et al., 2020). Positive controls are most studied in symbiotic relationships, including legume–rhizobia, arbuscular mycorrhizal fungi (AMF)–SLs, and actinorhizal plant–*Frankia* interactions; such communication establishment relies on both root signals and microbe signals. Root exudates containing antagonistic “signals” directly inhibit or kill (static or cidal) pathogenic microbes [through root exudate signals: phenolic compounds, non-volatile terpenoids, volatile terpenes, and sulfurous compounds (Rizaludin et al., 2021)] or possibly by suppressing the production of microbe signals back to plants. The defense mechanism of plants is set off by regulating root exudation after receiving the signal released from beneficial or pathogenic bacteria (Hernández-Calderón et al., 2018). Therefore, the behavior of plants, with regard to releasing negative signals, is generally regarded as self-protective in nature.

### 2.1 Mediation among neighboring plants

Root exudate chemical signals detect disturbances within the rhizosphere or surrounding environments and assist plants in adapting to and resisting the potential negative effects of unfavorable conditions (Zhalnina et al., 2018; Tian et al., 2020). Allelochemical signals are produced to improve plant robustness by reducing competition from neighboring plants (Wang et al., 2020). For instance, root exudates produced by barnyardgrass can induce the production of allelochemicals by rice, such as phenolic acids, that trigger induced systematic resistance and reduce weed presence, or acclimate the plant to weed competition stress (Zhang et al., 2018); the presence of barnyardgrass root exudate can be one of the most important factors contributing to phenolic synthesis in rice.

Root signals produced in crop rotation cultivation systems also play pivotal roles in the fitness and performance of next-generation plants. Hu et al. (2018) illustrated that benzoxazinoids (secondary metabolites produced by cereals, including maize) assist in the suppression of herbivore prevalence in the next generation for maize (*Zea mays* L., genotypes B73). In addition, high levels of organic acid anions released into soil by soybean and cowpea (genotypes TGm 1511 and IT89KD-391, respectively) improve P acquisition efficiency under P deficiency, and subsequently, organic P that accumulated in the previous legume crop residues contributes to enhance the following maize crop (Jemo et al., 2006). Legumes (e.g., faba bean) can release root exudates containing fixed N (e.g., NH_4_^+^ and amino acids) as a source of N for subsequent maize growth (Coskun et al., 2017). In return, an isoflavonoid signal produced by maize contributes to N availability and biomass yield improvement of faba bean. The above examples illustrate how, through signals, root exudates can facilitate belowground interactions and communications.

### 2.2 Communication between microbes and plants

Plant growth is affected by the rhizomicrobiome, the composition of which drives important elements of root activity, including root exudation of metabolites, and modulates signal transmission (Korenblum et al., 2020). Plants also contribute to the establishment of symbiotic relationships with associated microbes. In this process, root exudates as signals modulate the diversity and abundance of microbes in the rhizosphere (de Vries et al., 2020), which often happens under unfavorable environmental conditions (Morris and Moury, 2019; Rolfe et al., 2019). When under attack by the fungal pathogen *Fusarium oxysporum*, tryptophan is released from cucumber roots, acting as a signal recruiting *Bacillus amyloliquefaciens* to act against infection by the pathogen (Liu et al., 2017). In another example, tomato root exudates of lactic acid and hexanoic acid promoted the growth of the biocontrol strain of *Bacillus cereus*, reducing the infection rate of *Ralstonia solanacearum* (Wang et al., 2019). Exudates containing carbohydrate and organic acids can also increase the abundance of organic compound-degrading microorganisms (Lu et al., 2017; Zhalnina et al., 2018) so that their presence enhances the degradation of organic pollutants in the soil (Jia et al., 2016; Jia et al., 2018a). This is referred to as plant–soil feedback including both negative and positive feedback (Hu et al., 2018). Plants assemble the appropriate suite of beneficial microbes through changing the chemical composition of root exudates, thus improving plant resilience and fitness (Coskun et al., 2017). The type of root signals also varied significantly among plant species and ultimately determined the composition of the rhizomicrobiome (Wu et al., 2017; Luo et al., 2021). Rhizobia require the presence of isoflavonoid signals to activate nod genes, thus stimulating nodule formation by legumes (Coskun et al., 2017). Mycorrhizal fungi facilitate symbiotic interactions through strigolactone, a component of root exudates produced by plants (Akiyama and Hayashi, 2006; Ruyter-Spira and Bouwmeester, 2012). Therefore, rhizosphere communication is conducted and modulated by both host plants (root exudates) and microorganisms (Hortal et al., 2017; Mavrodi et al., 2021), contributing to the diversity and balance of the soil ecological web.

## 3 Identification and quantification of root exudates

The recognition of the critical role of root exudates in the rhizosphere and in the soil ecosystem that leads to the actions of secreted exudates has been studied more intensely over the last two decades. The methodologies for root exudate collection differ substantially among studies, due to the inaccessibility of the root system; the set of methods mainly include the following: (1) hydroponic systems, (2) soil-hydroponic mixing systems, and (3) soil growth and sampling. Detailed description used to collect root exudates and the comparisons among improved methods are provided in **Table 1**. It is no doubt that the sampling techniques improve with the understanding of the dynamics of related rhizosphere processes. The critical aspect of all three sets of approaches uses artificial systems to collect components of the exudate under axenic conditions to avoid overwhelming by chemicals in the soil. Despite the numerous studies modifying the techniques, the accuracy of the results obtained from these artificial conditions, compared with the ecologically relevant exudates released under complex rhizosphere conditions (e.g., field soil), in the context of the rhizosphere processes contributed to by exudates and the presence of other organisms, remains unclear. Therefore, it is doubtful that using root exudates produced under near-sterile conditions, instead of the complex natural rhizosphere, to explore the mode of action in the interaction between root exudates and its surrounding organisms accurately reflects normal composition and will produce “real world” results. Although a standard approach for root exudate sampling is yet to be exploited, studies that explore the mode of actions of root exudates could focus on plant responses under a set of parallel experiments with or without certain treatments.

**Table 1:** The approaches for the root exudates collection and improvements.

Methods	Description^1^	Improvements^2^	Reference
Hydroponic	1. Plants grown in the hydroponics setup with nutrient medium, then well rooted plant transferred to sterile distilled water; 2. Plant grown in hydroponic glass connected with a column containing XAD4 resin.	• Controlled growth conditions • Exudates collected from all types of roots • Less disturbance	Torabi et al. (2012); Kawasaki et al. (2018) Zhao et al. (2021);
Soil-Hydroponic	Plants grown in the soil until well rooted, then roots of intact plants washed to remove all remaining soil, then each individual plant transferred to a hydroponics system with sterile distilled water.	NA	Egle et al. (2003)
Soil growth	1. Plants grown in soil until well rooted, then roots washed and kept in glass cuvettes filled with sterile glass beads and C-free nutrient solution. Exudates collected by flushing each cuvette with a vacuum pump; 2. Lower part of roots was grown through a slit; 3. Plant grown in silica sand with glass bottle draining.	• Less disturbance • More simplification • High amount of root exudate • More selective	Phillips et al. (2008); Oburger et al. (2013); Luo et al. (2020)

^1^Numbers indicate the improved method following in order.

^2^By comparing the improved method in the description.

NA, not applicable.

Considering the interaction within all organisms, root exudate identification should not be separated from the rhizospheric organisms. In such situation, multi-omics approaches help to visualize the temporal–spatial distributions of root exudates and the associated microbiome. With regard to the beneficial aspects of each technique, genome sequences can firstly identify the specific traits of functional bacterial isolates involved in growth strategies, substrate uptake, and extracellular enzyme production related to fitness of host in the rhizosphere (Mönchgesang et al., 2016; Zhalnina et al., 2018). Then, an exometabolomics approach based on the principle of mass spectrometry further determines how the isolated microbes interact with root exudate metabolites (Silva and Northen, 2015). To study in detail metabolically active microbes that assimilate or respond to root exudates, an effective tool to at least partially understand which metabolites are plant- or microbe-derived is isotopic labeling, which can track the passage of an isotope through a reaction, metabolic pathway, or cell (Basu et al., 2011). Knowing the relationship between organisms, transcriptomics is next used to detect the relevant gene response to root exudate production (Yi et al., 2017; Zhang et al., 2020). Finally, proteomic analyses will provide important information regarding how gene-expression manipulations relate to specific proteins involved in modifying plant development and behavior, and how this impacts interactions with microbes, making them compatible or incompatible with the surroundings that the host is facing (Afroz et al., 2013; Zhang et al., 2020).

For structural elucidation of root exudate metabolites, collected exudate can also be fractionated by flash chromatography, purified by high-performance liquid chromatography–solid phase extraction (HPLC-SPE), and then subjected to nuclear magnetic resonance (NMR) analysis (Kalala et al., 2020). To examine the symbiotic relationship models between microbes and a single compound or signal, purified compounds can be used to test its bioactivities associated with microorganismal isolates in the rhizosphere. Improving the understanding of root exudate composition has the potential to lead to development of new techniques, in order to obtain plant metabolites on a large scale and could also extend our collective understanding regarding interactions of plants and associated microbes. These multi-omics can help us explore interactions among organisms within the holobiont, instead of focusing on single aspects (plants or microbial communities). Additionally, multiple technique applications could provide insight into the role(s) of these signal exchanges in inter-organismal interactions and the overall functioning and viability of a given holobiont.

## 4 Interaction of plant–microbial communities

### 4.1 Screening microbes’ signal on the base of root exudates

In the aspect of microbes involving root exudation production, pre-collection of exudates could be a part of screening to identify microbes that interact regularly with signals from plant roots and other associated microbes. Traditionally, microbial screening has been conducted in artificial growth media under sterile laboratory conditions to select microbes with potential key holobiont bioactivities. Microbes that are easily grown and that readily colonize plant roots are generally chosen for further analysis. There have been promising laboratory results from work in artificial media provided with approximately 100-fold higher concentrations of nutrients than generally occurs in the rhizosphere under field conditions (Lugtenberg et al., 2017). However, microbes isolated *in vitro* make up only about 1%–5% of total microbes (Pham and Kim, 2012). As the new update, Zhalnina et al. (2018) obtained 39 isolates representing approximately 10%–12% of the total bacterial community in the rhizosphere. It indicates that the true diversity of unculturable microorganisms in the rhizosphere is associated with *in situ* surroundings. Using rice as model, Edwards et al. (2015) found that methanogenic archaea were more enriched in the field (rice paddies) than in the greenhouse condition. The authors later verified that low levels of methane (CH_4_) production were detected under greenhouse growth (less rich methanogenic archaea) because methanogenic archaea cooperate with syntrophic partners to obtain H_2_ and formate for CH_4_ synthesis. In such cases, a novel genome-centric metatranscriptomics approach recently reported by Jia et al. (2018b); Hao et al. (2020), and Treu et al. (2016) can be used to effectively detect the low-abundant microbial members, specifically syntrophic bacteria, which cannot be analyzed based on isolates or enrichment cultures. This approach is useful for culture-independent identification of microbial involved in interaction with plant roots.

**Figure 1:**
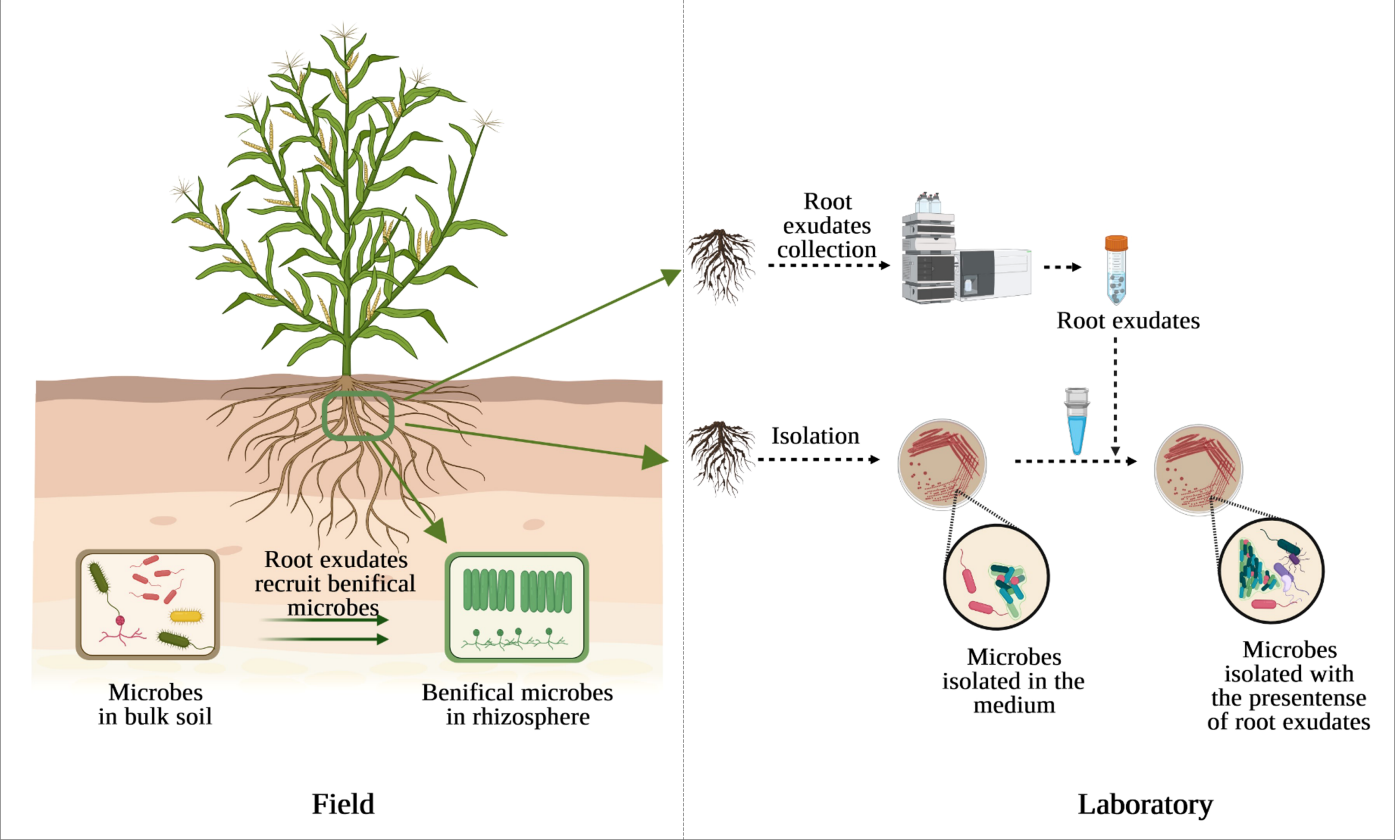
The presence of root exudates affects the diversity and abundance of beneficial microbes in the rhizosphere compared to bulk soil (left) and could also affect culturable microbes under laboratory condition (right).

On the other hand, screening or growing in pure culture is still an irreplaceable technique to study the physiological properties of an organism. One of the possible reasons for uncultured microbes is the absence of density-dependent cell signaling under laboratory conditions (Camilli and Bassler, 2006; Pham and Kim, 2012), while these microbes have probably co-evolved with plants for a protracted period. The exogenous root exudate malic acid secreted from banana and added into LB medium induced the chemotactic response and biofilm formation of the *B. amyloliquefaciens* NJN-6 (Yuan et al., 2015). Malic acid released from tomato and citric acid detected in cucumber exudates also induced the motility of *Paenibacillus polymyxa* SQR-21 (Ling et al., 2011) and *Bacillus amyloliquefaciens* SQR9 (Zhang et al., 2014), respectively, thereby contributing to preferential colonization into roots. These findings suggest that plant roots have profound influence on the capacity of different rhizosphere colonization of soil bacteria (da Rocha et al., 2014). Therefore, providing microbes with pre-collected plant root exudates may allow them to receive right signals or improve the growth rate and colonization efficiency of microbes on non-host plant species (**Figure 1**).

### 4.2 Coevolution of plant–microbial communities

Plant evolution has always involved microorganisms, including in the form of important organelles (mitochondria and chloroplasts), developed through endosymbiosis of prokaryotes leading to a rigid and completely obligatory mutualism interaction present in all terrestrial plants (Lyu et al., 2021a; Lyu et al., 2021b). Plant evolution is meaningfully driven by interactions with symbiotic microbes (Delaux and Schornack, 2021), where the presence of the microbe substantially enhances the fitness of the plant. Root exudates play a critical role in the establishment of symbiosis relationships between plants and microorganisms. In the case of the legume nitrogen-fixing symbiosis, isoflavonoids produced by legume plants induce rhizobia to release lipo-chitooligosaccharides (LCOs) as signals back to the legume, initiating nodule formation, leading to nitrogen fixation; in addition, LCOs enhance stress resistance in legumes and also a wide range of non-legume plants, contributing to critical modifications of the physicochemical properties of target plants, thus emphasizing the importance of symbiosis establishment with legumes and the regulation of abiotic stress tolerance in many other plant groups (Lyu et al., 2020). In contrast, AMF can establish symbiotic relationships with 80% of higher plants, which rely on the fungal hyphae to assist in acquiring nutrients; AMF require contact with the host plant root to finish their life cycle (Wipf et al., 2019). In this symbiosis, specific root exudates (strigolactones and flavonoids) produced by the host can communicate with AMF to facilitate the AMF symbiosis *via* stimulation of the Myc Factor production to set the stage for the establishment of the symbiosis that will enhance the availability of nutrients for host plants in, for instance, maize–legume intercropping systems (Coskun et al., 2017).

The establishment of microbial communities on plants is influenced by the plant compartment (Bai et al., 2015) and host species (Ofek-Lalzar et al., 2014; Tkacz et al., 2015) and soils (del Pilar Martínez-Diz et al., 2019; Edwards et al., 2019). Plants are predominantly photosynthetic eukaryotes of the kingdom Plantae, which are commonly defined as C3, C4, and CAM (Crassulacean Acid Metabolism) according to the CO_2_ fixation mechanism (Sivaram et al., 2020). The photosynthetic carbon fixation pathways are critical for plants to convert CO_2_ into what they need for their growth and development (Heyneke and Fernie, 2018). Moreover, different types of plants ultimately release different quantities and qualities of compounds into the rhizosphere as root exudates. Some studies reported that a greater variety of dominant organic acids and sugars are released by C3 plants (Zhu et al., 2008; Meyer et al., 2010; Vranova et al., 2013), but a higher quantity of root exudates is secreted by C4 plants, as they can fix carbon more rapidly through greater photosynthetic rates (Bledsoe et al., 2020). Thus, the different compositions (i.e., carbohydrates, organic acids, and amino acid) of root exudates could affect the assemblage of associated microorganisms. For instance, meta-analysis has shown that there are more AMFs in the rhizosphere when C4 plants are grown in a soil than when C3 plants are in the same soil (Pirttilä et al., 2021). Prior studies also demonstrated that C4 plants recruit a higher abundance of the polycyclic aromatic hydrocarbon degrading bacterial community than C3 plants because photosystems of C3 and C4 led to differences in root secretions (Sivaram et al., 2020). However, C4 plants may be less efficient at photosynthesis under the higher CO_2_ levels of the near future. The composition of root exudates released by C3 and C4 plants may change, such as increases in organic acid production under higher atmospheric CO_2_ levels (Xiong et al., 2019). For instance, the abundance of organic acids, oxalate, citrate, and malate is increased in root exudates under elevated CO_2_ (Sánchez-Carrillo et al., 2018). Interestingly, the root exudation of organic C from C_3_ and C_4_ plants can be changed by the soil nutrient status, further affecting rhizosphere bacterial community structure (Carvalhais et al., 2011; Wu et al., 2012; Bledsoe et al., 2020). Host species with CAM metabolism can also assemble the unique bacterial, archaeal, and fungal communities in the rhizosphere (Citlali et al., 2018). Therefore, the plant–microbe interactions in a given environment is mainly determined by the C fixation or plant photosynthesis modes and the allocation patterns of C released by the plants (Cregger et al., 2021). The rate of C fixation affects the component of root exudates, which contributes to the network of plant–microbe–plant and plant–plant communications and assembly of the unique holobiont; thus, they can play a role in the establishment of symbiotic relationships and thereby contribute to plant physiology, development, and biomass production. However, the role of root exudates has been elucidated in only a few of these symbioses, encompassing only a few specific plant species. Future investigations of these interactions, involving a range of plant types, may uncover new modes of molecular symbiotic interaction.

## 5 Conclusion

Root exudate is a key element of plant homeostasis, in part through playing a key role in communication between aboveground and belowground elements of plants. Root signals affect interactions with the associated phytomicrobiome, leading to a functional and effective holobiont that benefits plant growth. However, there are certainly still unknown signals in inter-organismal communications, produced by both plants and microbes. Discovering and exploring more signals will extend our understanding of plant–plant and plant–microbe interactions. Moreover, exploiting new techniques for collecting plant metabolites will provide access to various inter-organismal signals on a large scale. In the long term, agricultural production will be able to take full advantage of the phytomicrobiome by manipulating plant root signal production. Importantly, plant C-fixation strategy is a major component driving rhizospheric development and maintenance. Evaluating the underground impact of biodesign programs leveraging C-fixation engineering in plants will thus be of great importance. All bioprospecting under laboratory conditions aims to identify the potential beneficial microbes that then could be efficiently employed as a sustainable strategy to improve crop plant resilience and overall crop productivity. One application of root exudates could be as bio-inoculants to trigger the signaling between plants and microbes and also recruit beneficial microbes, as well as remove contaminants from the soil, thus enhancing the crop yield and biomass. Thus, the identification of components of exudates and understanding the mechanisms by which they regulate rhizomicrobiome networks should be considered an important line of future research.

## 6 Outstanding questions

- Are there one or more specific root exudate compounds/materials that cause/allow specific activities in plant-associated bacteria, including the ability to grow *in vitro*? If so, are these specific root exudates spontaneous or are they induced?
- What are the effects of stressful growth conditions on root exudate production? To what extent is secondary metabolite production in other parts of plants changed with the root exudate?
- Were root exudates involved in the evolution of phytomicrobiome? If so, what does this tell us about the holobiont?

## Author contributions

DL gathered literature and prepared the manuscript. DS provided feedback and oversaw progression of the manuscript. All authors gave final approval for publication and agreed to be held accountable for the work contained therein.
